# Supervised Manifold Learning Based on Multi-Feature Information Discriminative Fusion within an Adaptive Nearest Neighbor Strategy Applied to Rolling Bearing Fault Diagnosis

**DOI:** 10.3390/s23249820

**Published:** 2023-12-14

**Authors:** Hongwei Wang, Linhu Yao, Haoran Wang, Yu Liu, Zhiyuan Li, Di Wang, Ren Hu, Lei Tao

**Affiliations:** 1 Center of Shanxi Engineering Research for Coal Mine Intelligent Equipment, Taiyuan University of Technology, Taiyuan 030024, China; wanghongwei01@tyut.edu.cn (H.W.); wanghaoran01@tyut.edu.cn (H.W.); taoted@163.com (L.T.); 2 College of Mechanical and Vehicle Engineering, Taiyuan University of Technology, Taiyuan 030024, China; liuyu0018@link.tyut.edu.cn (Y.L.); zhiyuanl9@163.com (Z.L.); wdafzj@126.com (D.W.); huren0009@link.tyut.edu.cn (R.H.)

**Keywords:** intelligent fault diagnosis, adaptive nearest neighbor strategy, exponential linear kernel function, discriminative fusion of multi-feature information

## Abstract

Rolling bearings are a key component for ensuring the safe and smooth operation of rotating machinery and are very prone to failure. Therefore, intelligent fault diagnosis research on rolling bearings has become a crucial task in the field of mechanical fault diagnosis. This paper proposes research on the fault diagnosis of rolling bearings based on an adaptive nearest neighbor strategy and the discriminative fusion of multi-feature information using supervised manifold learning (AN-MFIDFS-Isomap). Firstly, an adaptive nearest neighbor strategy is proposed using the Euclidean distance and cosine similarity to optimize the selection of neighboring points. Secondly, three feature space transformation and feature information extraction methods are proposed, among which an innovative exponential linear kernel function is introduced to provide new feature information descriptions for the data, enhancing feature sensitivity. Finally, under the adaptive nearest neighbor strategy, a novel AN-MFIDFS-Isomap algorithm is proposed for rolling bearing fault diagnosis by fusing various feature information and classifiers through discriminative fusion with label information. The proposed AN-MFIDFS-Isomap algorithm is validated on the CWRU open dataset and our experimental dataset. The experiments show that the proposed method outperforms other traditional manifold learning methods in terms of data clustering and fault diagnosis.

## 1. Introduction

With the rapid development of industrial intelligence, intelligent fault diagnosis technology is playing an increasingly important role in maintaining the health of mechanical equipment and ensuring its safe and stable operation [1]. Rolling bearings, as critical components of rotating machinery, often operate in harsh and variable environments, such as at high speed and under heavy loads, making them prone to wear and failure and leading to severe mechanical accidents [2]. To avoid significant economic losses and casualties, research on intelligent fault diagnosis methods for rolling bearings is particularly important [3]. Fault diagnosis is fundamentally a pattern recognition problem, and analyzing and processing vibration signals during the operation of rolling bearings is an effective approach for diagnosing faults in rotating machinery. One of the most crucial aims is to break the “curse of dimensionality” and extract low-dimensional features with high sensitivity [4,5,6].

To enhance the quality of feature extraction and address the severe “curse of dimensionality” issue at the current stage, manifold learning algorithms have emerged. Classical manifold learning algorithms mainly include Principal Component Analysis (PCA), Independent Component Analysis (ICA), Linear Discriminant Analysis (LDA), and Multidimensional Scaling (MDS). However, these algorithms are primarily used for linear dimensionality reduction and may not be suitable for high-dimensional nonlinear vibration data from rolling bearings [7,8].

In the year 2000, Joshua B. Tenenbaum and Sam T. Roweis [9,10] proposed two classic nonlinear manifold learning dimensionality reduction algorithms, Isometric Feature Mapping (Isomap) and Locally Linear Embedding (LLE), in science. Since then, manifold learning algorithms have been extensively researched by researchers and have gradually become a research hotspot in the fields of dimensionality reduction and pattern recognition [9,10,11]. Based on their different mathematical assumptions, manifold learning algorithms are divided into two major categories: locally preserving embedding methods and globally preserving embedding methods. Laplacian Eigenmaps (LE) [12], LLE [10], Hessian-based Locally Linear Embedding (HLLE) [13], and Local Tangent Space Alignment (LTSA) [14] are considered local preserving embedding methods in manifold learning, while Isomap [8], Diffusion Maps (DM) [2], and t-Stochastic Neighbor Embedding (t-SNE) [15] are regarded as globally preserving embedding methods. However, regardless of the manifold learning algorithm’s mathematical assumptions, there is a bottleneck in selecting neighboring points [6,11].

To address the sensitivity of neighbor point selection in manifold learning algorithms, Zhenyue Zhang et al. conducted research from two perspectives: adaptive neighbor selection and the interaction between manifold curvature and sampling density [16]. They explored methods for constructing nonlinear low-dimensional manifolds from high-dimensional space samples, providing directions for subsequent researchers. Chuang Sun et al. conducted research from the perspective of adaptive neighbors and used the kernel sparse representation method to select sample neighbors and reconstruct the weights of the neighbor graph for the LLE algorithm [17]. Yan Zhang et al. integrated nonnegative matrix factorization with sparsity constraints based on the work in reference [17] and applied it to the LLE algorithm to jointly minimize the neighborhood reconstruction error on the weight matrix [18]. All of these methods use sparsity constraints to select neighbor points, but they perform relatively averagely when the data contain noise points and outliers.

To address this issue, Yunlong Gao et al. proposed a discriminant analysis based on the reliability of local neighborhoods, enhancing the performance of effective samples in low-dimensional space and filtering the interference of outliers, thereby improving the dimensionality reduction ability [19]. Jing An et al. introduced an adaptive neighborhood-preserving discriminant projection model [20]. By updating sparse reconstruction coefficients, the adverse effects of noise and outliers on the dimensionality reduction were mitigated, enhancing sample clustering. Jiaqi Xue et al. proposed a locally linear embedding method by applying an adaptive neighbor strategy, preserving more original information when embedding high-dimensional data manifolds into low-dimensional space and achieving better clustering results [11]. It can be observed that various discrimination methods have been widely applied in manifold learning models. Most of these adaptive neighbor strategies and discrimination methods are applied in locally preserving embedding methods of manifold learning algorithms, while their application in globally preserving embedding manifold learning algorithms is limited, especially in unsupervised learning models.

To address the aforementioned issue, incorporating label information into the algorithm’s supervised learning mode can further enhance its clustering capability. Ratthachat Chatpatanasiri et al. proposed a general framework for manifold learning semi-supervised dimensionality reduction, providing research directions for subsequent researchers [21]. Jing Wang et al. proposed a semi-supervised manifold alignment algorithm that utilizes sample points and their corresponding relationships to construct connections between different manifolds [22]. Zohre Karimi et al. introduced a novel hierarchical spatial semi-supervised metric learning approach, integrating local constraints and information-theoretic nonlocal constraints to better represent the smoothness assumption of multiple manifolds using the metric matrix [23]. Mingxia Chen et al. proposed a robust semi-supervised manifold learning framework applied in locally preserving embedding manifold learning algorithms to eliminate adverse effects caused by noise points [24]. Ding Li et al. derived an extension of a semi-supervised manifold regularization algorithm for classification tasks, optimizing the algorithm’s performance on multi-class problems using weighted strategies [25]. Jun Ma et al. proposed a secure semi-supervised learning framework, using both manifold and discriminant regularization to mitigate the influence of unlabeled points and boundary points in the pattern recognition process [26]. However, the impact of unlabeled points and boundary points in the semi-supervised learning mode on the model’s clustering and classification capabilities remains unresolved.

Therefore, researchers have applied supervised learning modes to manifold learning models, which, compared to manifold learning models under the semi-supervised learning mode, demonstrate stronger robustness in handling classification problems [27,28,29]. However, current research methods are limited to dimensionality reduction and fault diagnosis tasks on a single feature space within the manifold learning model. The feature information in the data is singular and incomprehensive.

In summary, manifold learning methods have been widely applied in the fields of dimensionality reduction and fault diagnosis. However, they still have limitations. The issues of neighbor point selection in manifold global preservation embedding, the influence of data outliers on clustering effectiveness, and the singular and incomplete feature information contained in the data have not been fully addressed. To address these problems and build upon existing research, this paper proposes a supervised manifold learning approach for rolling bearing fault diagnosis based on the discriminative fusion of multiple pieces of feature information using an adaptive nearest neighbor strategy.

The main contributions of this paper are summarized as follows:

1. Propose an adaptive neighbor selection strategy that amalgamates the Euclidean distance and cosine similarity measures. This strategy systematically computes both the distance and angular information among neighboring points, utilizing the metric mean as the discriminant criterion. By configuring the preset neighboring points as the criterion object, it dynamically adjusts the proximity graph to refine the local structure of the manifold. This process is aimed at enhancing the precision of the manifold space depiction and local feature representation and reducing the adverse effects of data outliers on clustering performance.
2. Propose three methods for transforming feature spaces and extracting spatial feature information and space information. Notably, this paper proposes a unique form of the kernel function, the exponential linear kernel function, which serves to project data into a novel kernel Hilbert space. Concurrently, this function is employed as the nonlinear discriminant mapping function in the Supervised Version of the Isometric Feature Mapping (S-Isomap) algorithm, thus providing a distinct representation of data in the manifold space. The extracted feature information, originating from diverse kernel Hilbert spaces and manifold spaces, ensures the intricate and sensitive nature of the features.
3. Propose a fault diagnosis algorithm model for rolling bearings by employing a supervised learning paradigm. Under the adaptive neighbor selection strategy, features from different spaces are merged which are both sensitive and complex to form a multi-space metric matrix. This matrix is designed to encapsulate substantial multi-space feature information, enabling its fusion with machine learning classifier algorithms to facilitate fault diagnosis.

The structure of this paper is organized as follows: Section 2 introduces the foundational manifold learning algorithms Isomap and S-Isomap along with their relevant theories. Section 3 presents the proposed supervised manifold learning method involving an adaptive neighbor strategy, the extraction of multi-space feature information, and the discriminative fusion of multiple pieces of feature information. Section 4 conducts an evaluation of the model’s clustering and classification capabilities, analyzing and comparing the proposed approach in this paper with traditional manifold learning methods from both qualitative and quantitative perspectives. Finally, Section 5 provides a comprehensive summary of the entire paper.

## 2. Related Work

The core idea of manifold learning is based on the manifold assumption, which posits that data are distributed on a smooth low-dimensional manifold embedded in a high-dimensional space. Traditional manifold learning algorithms such as Isomap, LLE, and LTSA aim to find the embedded low-dimensional manifold within a high-dimensional space [30]. This can be described mathematically as finding the mapping process, f, such that $f:\;X={\left[x_{1},x_{2},\cdots ,x_{N}\right]}^{T}\in {\mathbb{R}}^{N\times D}\to Y={\left[y_{1},y_{2},\cdots ,y_{N}\right]}^{T}\in {\mathbb{R}}^{N\times d}\left(d\ll D\right)$, where $x_{i}$ denotes the sample in a high-dimensional space, X; $y_{i}$ is the mapping of sample $x_{i}$ in a low-dimensional space, Y; *N* is the number of data points; *D* is the number of high-dimensional features; and *d* is the number of low-dimensional features.

### 2.1. Isometric Feature Mapping (Isomap)

Isomap, as one of the most traditional manifold learning algorithms, operates on the principle of preserving the global geometric properties of the intrinsic low-dimensional manifold to obtain a low-dimensional representation of the data. Isomap modifies the measurement method used in MDS, where the Euclidean distance describes the relationship between two data points, to a method based on the geodesic distance on the manifold [9]. The Isomap algorithm is as follows:

(1) Calculate the Euclidean distance, $d_{E}\left(x_{i},x_{j}\right)$, between any two data points. Then, use k-nearest neighbors (k-NNs) from the number information of samples or ε-nearest neighbors (ε-NNs) from the distance information of samples to construct a simple undirected nearest neighbor graph, *G*. If $x_{i}$ and $x_{j}$ are neighbors, then connect $x_{i}$ and $x_{j}$ in *G* and assign a weight, $d_{E}\left(x_{i},x_{j}\right)$, to the edge.
(2) Based on the edges of the simple undirected nearest neighbor graph, G, use Dijkstra’s algorithm or Floyd’s algorithm to calculate the geodesic distances, $d_{G}\left(x_{i},x_{j}\right)$.
(3) To establish the low-dimensional embedded manifold coordinates, Y, based on an objective function, the typical objective function for Isomap can be expressed as follows:
  (1) $$L_{Iso}=\underset{Y}{\min}\sqrt{\sum_{i,j}{\left(d_{G}\left(x_{i},x_{j}\right)-d_{E}\left(y_{i},y_{j}\right)\right)}^{2}}$$
  where $d_{E}\left(y_{i},y_{j}\right)$ represents the Euclidean distance of the mapping of the Euclidean distance, $d_{E}\left(x_{i},x_{j}\right)$, in a high-dimension space.
(4) To compute the low-dimensional embedded manifold coordinates, Y, use MDS. To be specific, let $D_{G}^{2}=\left[d_{G}^{2}\left(x_{i},x_{j}\right)\right]$ and set $\tau \left(D_{G}^{2}\right)=-HD_{G}^{2}H/2$, where $H=I-ee^{T}/N$ is the centered matrix and *e* is the column vector of all the matrices. Perform an eigenvalue decomposition on $\tau \left(D_{G}^{2}\right)$ to obtain the low-dimensional embedded manifold coordinates $Y={\left[y_{1},y_{2},\cdots ,y_{N}\right]}^{T}={\left[\sqrt{\lambda_{1}}u_{1},\sqrt{\lambda_{2}}u_{2},\cdots ,\sqrt{\lambda_{d}}u_{d}\right]}^{T}$, where $\lambda_{p}$ and $u_{p}$ denote the $p^{th}$ dominant eigenvalue and its eigenvector, respectively [30].

### 2.2. Supervised Version of Isometric Feature Mapping (S-Isomap)

The S-Isomap algorithm incorporates label information from the data as prior knowledge to guide the dimensionality reduction process. Utilizing data labels as discriminant information, the data are initially divided into a true nearest neighbor set, $S^{+}$, and a pseudo nearest neighbor set, $S^{-}$, which guides the discriminant manifold learning process. $S^{+}$ and $S^{-}$ are defined as follows:

(2) $$S^{+}=\left\{\left(x_{i},x_{j}\right)\left|L\left(x_{i}\right)=L\left(x_{j}\right),x_{j}\in N\left(x_{i}\right)\right.\right\}$$

(3) $$S^{-}=\left\{\left(x_{i},x_{j}\right)\left|L\left(x_{i}\right)\neq L\left(x_{j}\right),x_{j}\in N\left(x_{i}\right)\right.\right\}$$

where $L\left(x_{i}\right)$ denotes the class label of $x_{i}$ and $N\left(x_{i}\right)$ are the neighborhood sets of $x_{i}$. Next, based on $S^{+}$ and $S^{-}$, we construct the true nearest neighbor graph, $G^{+}$, and pseudo nearest neighbor graph, $G^{-}$. Then, the objective of the S-Isomap algorithm is to preserve the intrinsic geometric structure of the data within the same class and to separate different classes by optimizing the following objective function:

(4) $$L_{SIso}^{S+}=\underset{Y}{\min}\sqrt{\sum_{(x_{i},x_{j})\in S^{+}}{\left(d_{E}\left(y_{i},y_{j}\right)-d_{G}\left(x_{i},x_{j}\right)\right)}^{2}}$$

(5) $$L_{SIso}^{S-}=\underset{Y}{\max}\sqrt{\sum_{(x_{i},x_{j})\in S^{-}}{\left(d_{E}\left(y_{i},y_{j}\right)-d_{G}\left(x_{i},x_{j}\right)\right)}^{2}}$$

To find the optimal solution for the objective function mentioned earlier, the S-Isomap algorithm rescales the metric (Euclidean distance) between two data points as follows:

(6) $$D_{E}\left(x_{i},x_{j}\right)=\left\{\begin{array}{cc} \sqrt{1-e^{\frac{-d_{E}^{2}\left(x_{i},x_{j}\right)}{\beta }}} & L\left(x_{i}\right)=L\left(x_{j}\right)\\ \sqrt{e^{\frac{-d_{E}^{2}\left(x_{i},x_{j}\right)}{\beta }}}-\alpha & L\left(x_{i}\right)\neq L\left(x_{j}\right) \end{array}\right.$$

where β is a parameter with relation to the scale of a dataset and α is a parameter which can adjust the intra-class dissimilarity sensitively. The two values α and β are usually determined empirically. Finally, we compute the low-dimensional embedded manifold coordinates, Y, using MDS [30].

## 3. AN-MFIDFS-Isomap Reduction

### 3.1. Adaptive Nearest Neighbor Strategy

The value of the nearest neighbor count, k, serves as a hyperparameter in manifold learning algorithms, determining the size of local regions within the manifold. A larger k value may smooth or eliminate small-scale structures within the manifold. Conversely, a smaller k value might incorrectly partition a continuous manifold into disjoint submanifolds, thereby affecting the accuracy of the algorithm in approximating the global geometric structure and computing the metric matrix during the process. The appropriate choice of k is crucial to balance between capturing local details and preserving the overall manifold structure accurately.

**Figure 1:**
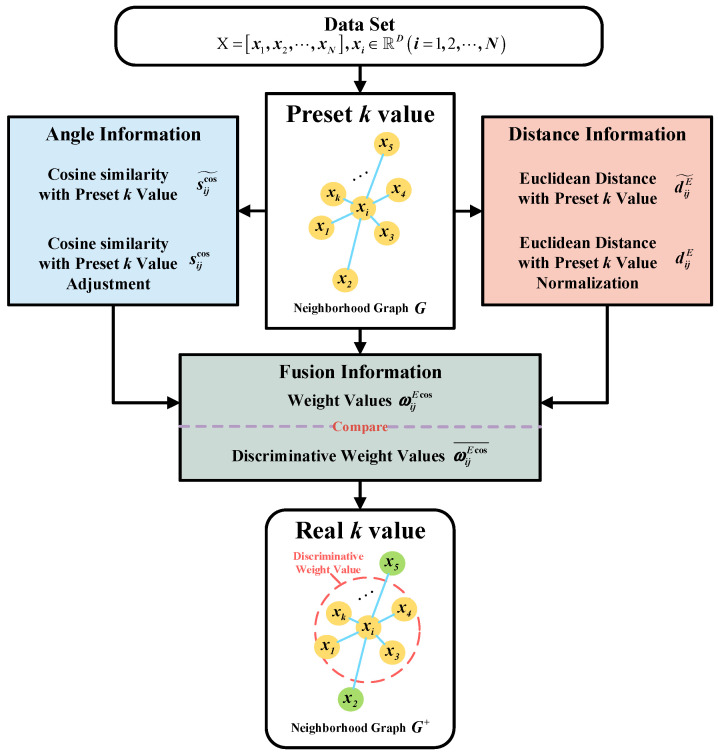
Flowchart of adaptive neatest neighbor strategy.

In this study, we introduce an adaptive nearest neighbor strategy by integrating both distance and angular information among data points. This strategy incorporates the concepts of initial nearest neighbors, pseudo nearest neighbors, and true nearest neighbors. By combining the data distance and angular information, we calculate the edge weights between data points, which are used to construct a simple undirected nearest neighbor graph for true nearest neighbors. The adaptive nearest neighbor strategy can effectively suppress the adverse effect of data outliers on clustering performance. The algorithm’s workflow is illustrated in Figure 1, and detailed steps of the algorithm are outlined as follows:

(1) Define the initial nearest neighbor count, k, and construct a simple undirected nearest neighbor graph, G;
(2) Construct the cosine similarity matrix for the data. Compute the cosine similarity between two data points using the following equation to obtain angular information:
  (7) $$\begin{array}{cc} \tilde{S_{ij}^{\cos}}={\left[\tilde{s_{ij}^{\cos}}\right]}_{N\times k}=\left[\frac{\langle x_{i},x_{j}\rangle }{{\left\|x_{i}\right\|}_{2}\cdot {\left\|x_{j}\right\|}_{2}}\right] & x_{j}\in N\left(x_{i}\right) \end{array}$$
  where $\langle \cdot ,\cdot \rangle$ denotes the vector’s inner product, ${\left\|\cdot \right\|}_{2}$ denotes the L2-norm of the vector, and ${\left[\cdot \right]}_{N\times k}$ denotes the shape of the matrix, which is N×k.
(3) Compute the normalized cosine similarity matrix by normalizing the angular information using the following equation:
  (8) $$S_{ij}^{\cos}={\left[s_{ij}^{\cos}\right]}_{N\times k}=\left[\log_{2}\left(1.5-0.5\tilde{s_{ij}^{\cos}}\right)\right]$$
  where $1\leq \left(1.5-0.5\tilde{s_{ij}^{\cos}}\right)\leq 2$.
(4) Construct the Euclidean distance matrix for the data. Compute the Euclidean distance between two data points using the following equation to obtain distance information:
  (9) $$\begin{array}{cc} \tilde{D_{E}}={\left[\tilde{d_{ij}^{E}}\right]}_{N\times k}=\left[{\left\|x_{i}-x_{j}\right\|}_{2}\right] & x_{j}\in N\left(x_{i}\right) \end{array}$$
(5) Compute the normalized Euclidean distance matrix by normalizing the distance information using the following equation:
  (10) $$D_{E}={\left[d_{ij}^{E}\right]}_{N\times k}=\left[\frac{\tilde{d_{ij}^{E}}-\min\left(\tilde{d_{i\cdot }^{E}}\right)}{\max\left(\tilde{d_{i\cdot }^{E}}\right)-\min\left(\tilde{d_{i\cdot }^{E}}\right)}\right]$$
  where $\tilde{d_{i\cdot }^{E}}$ represents all elements of row *i*, $\tilde{d^{E}}$.
(6) Construct the weight matrix by integrating the angular and distance information. Reassign the edge weights in the simple undirected nearest neighbor graph, G, using the following equation to fuse the angular and distance information:
  (11) $$W_{ij}^{E\cos}={\left[\omega_{ij}^{E\cos}\right]}_{N\times k}=\left[s_{ij}^{\cos}+d_{ij}^{E}\right]$$
(7) Compute the weight discrimination criterion. Calculate the average weight for each data point using the following equation to serve as the discrimination criterion:
  (12) $$\bar{W_{ij}^{E\cos}}={\left[\bar{\omega_{ij}^{E\cos}}\right]}_{N}=\left[\frac{1}{k+1}\sum_{j=1}^{k}\omega_{ij}^{E\cos}\right]$$

If $\omega_{ij}^{E\cos}\leq \bar{\omega_{ij}^{E\cos}}$, then $x_{j}$ is considered a true nearest neighbor of $x_{i}$. Conversely, if $\omega_{ij}^{E\cos}>\bar{\omega_{ij}^{E\cos}}$, then $x_{j}$ is considered a pseudo nearest neighbor of $x_{i}$. This process leads to dynamically obtaining the true nearest neighbor values, $k^{+}$, a simple undirected nearest neighbor graph, $G^{+}$ for true nearest neighbors, and a new set of rules for calculating the metric matrix.

The adaptive neighbor strategy proposed in this section offers a novel approach for dynamically selecting neighboring points in manifold learning. Essentially, it seamlessly combines k-NNs and ε-NNs by integrating the angular and distance information to derive weights. These weights are compared with the average weight, ultimately resulting in the construction of a simple undirected nearest neighbor graph, $G^{+}$, for true nearest neighbors.

### 3.2. Multi-Space Transformation and Feature Extraction

#### 3.2.1. Kernel Trick

The data matrix in the original space, denoted as $X={\left[x_{1},x_{2},\cdots ,x_{N}\right]}^{T}$, consists of N observation vectors of the data [31]. The kernel trick is a mathematical technique that utilizes a kernel function to map the data from the original space to a higher-dimensional Hilbert space, as follows:

(13) Φ:X→H xi↦ϕxi

where $\phi \left(\cdot \right)$ denotes the mapping of the kernel function.

The data matrix in the higher-dimensional Hilbert space is computed through the inner product of the observation vectors using the kernel function. This can be seen as the metric matrix in the higher-dimensional Hilbert space, as follows:

(14) $$k\left(x_{i},x_{j}\right)=\langle \phi \left(x_{i}\right)\cdot \phi \left(x_{j}\right)\rangle =\phi {\left(x_{i}\right)}^{T}\phi \left(x_{j}\right)$$

#### 3.2.2. The First Spatial Transformation and Feature Extraction

In this section, we use K-Isomap as the first spatial transformation method. This method considers the double centering process in Isomap as a kernel trick mapping process [32]. Therefore, the objective function is as follows:

(15) $$L_{KIso}=\underset{Y}{\min}\sqrt{\sum_{i,j}{\left(k\left(x_{i},x_{j}\right)-d_{E}\left(y_{i},y_{j}\right)\right)}^{2}}$$

To obtain the optimal solution for the objective function mentioned above, the problem is transformed into constructing a space metric matrix that better adheres to the manifold assumption. K-Isomap considers the geodesic distance matrix of the original data as the metric matrix during the double centering process. To ensure the semi-positive definiteness of the double-centered matrix, the Mercer kernel matrix method [33] is utilized to construct the first spatial metric matrix. The spectral radius of this matrix is computed to represent the first piece of spatial feature information.

Compared to the classical Isomap algorithm, the K-Isomap algorithm exhibits stronger robustness. The specific process of the algorithm is as follows:

(1) Using the traditional Isomap algorithm, construct a simple undirected nearest neighbor graph, G. Then, calculate the geodesic distance, $d_{G}\left(x_{i},x_{j}\right)$, between data points $x_{i}$ and $x_{j}$ using the shortest path algorithm and define the matrix $D_{G}^{2}=\left[d_{G}^{2}\left(x_{i},x_{j}\right)\right]$ and $\tau \left(D_{G}^{2}\right)=-HD_{G}^{2}H/2$;
(2) Construct the following block matrix:
  (16) $$\left[\begin{array}{cc} 0 & 2\tau \left(D_{G}^{2}\right)\\ -I & -4\tau \left(D_{G}\right) \end{array}\right]$$
  where 0 denotes the full-zero matrix and *I* denotes the unit matrix. Next, calculate its spectral radius, $\rho_{1}^{*}=\max\left(\lambda_{1},\lambda_{2},\cdots ,\lambda_{2N}\right)$, where $\lambda_{i}$ are all the eigenvalues of the above block matrix;
(3) Construct the Mercer kernel matrix as the first spatial metric matrix using the following equation:
  (17) $$K_{1}=\tau \left(D_{G}^{2}\right)+2\rho_{1}^{m}\tau \left(D_{G}\right)+\frac{1}{2}{\left(\rho_{1}^{m}\right)}^{2}H$$
  where the parameter $\rho_{1}^{m}\geq \rho_{1}^{*}$;
(4) Perform eigenvalue decomposition and select the top $\lambda_{1}$ eigenvalues’ corresponding to the eigenvectors $\Gamma_{1}$ as the first piece of spatial feature information for $K_{1}$. Calculate the spectral radius $\rho_{1}$ of the first spatial metric matrix, $K_{1}$, to represent the first piece spatial information.

#### 3.2.3. The Second Spatial Transformation and Feature Extraction

In the current manifold learning algorithms, widely used kernel functions include the Gaussian kernel function and the linear kernel function. The Gaussian kernel function is suitable for addressing complex nonlinear problems; however, it is highly affected by hyperparameter noise. On the other hand, the linear kernel function is suitable for addressing issues related to stability and poor generalization capabilities but does not effectively solve complex nonlinear problems [34]. In this section, we propose an exponential linear kernel function that combines the advantages of the above two kernel functions. The expression for the exponential linear kernel function is as follows:

(18) $$k_{Elinear}\left(x_{i},x_{j}\right)=e^{-\left(\varpi d_{E}\left(x_{i},x_{j}\right)+b\right)}$$

where the parameter ϖ>0 denotes the linear weight and the parameter *b* denotes the offset coefficient. A detailed proof of the positive semi-definite property of the constructed kernel matrix for the exponential linear kernel function is provided in Section 6, Appendix A.

In this section, we employ the exponential linear kernel function to construct the second space. We utilize the Mercer kernel matrix method to construct the metric matrix for the second space. The spectral radius of this matrix is then calculated to represent the second piece of spatial feature information. The specific steps of the algorithm are as follows:

(1) Apply the traditional Isomap algorithm to construct a simple undirected nearest neighbor graph, G, and calculate the Euclidean distance, $d_{E}\left(x_{i},x_{j}\right)$;
(2) Construct the exponential linear kernel matrix as follows, based on Equation (17):
  (19) $$K_{Elinear}=\left[k_{Elinear}\left(x_{i},x_{j}\right)\right]=\left[e^{-\left(\varpi d_{E}\left(x_{i},x_{j}\right)+b\right)}\right]$$
  Then, perform an eigenvalue decomposition and select the top $\lambda_{2}$ eigenvalues’ corresponding to the eigenvectors $\Gamma_{2}$ as the second piece of spatial feature information for $K_{Elinear}$. Let the spectral radius $\rho_{2}$ represent the second piece of spatial information.

#### 3.2.4. The Third Spatial Transformation and Feature Extraction

In this section, we propose a KS-Isomap manifold learning algorithm as the third space transformation method. The KS-Isomap algorithm utilizes data label information to guide the construction of the manifold and employs kernel tricks to map the metric matrix to a more abstract feature space. The KS-Isomap algorithm combines the robustness of the K-Isomap algorithm with the discriminative power of the S-Isomap algorithm.

The KS-Isomap algorithm follows the structure of the S-Isomap algorithm. It utilizes label information to guide the construction of the manifold. Simultaneously, it replaces the original metric matrix with the geodesic distance matrix and maps the geodesic distance matrix to the kernel space. Therefore, the objective function of the algorithm is represented by the following equation:

(20) $$L_{KSIso}^{S+}=\underset{Y}{\min}\sqrt{\sum_{(x_{i},x_{j})\in S^{+}}{\left(d_{G}\left(y_{i},y_{j}\right)-k\left(x_{i},x_{j}\right)\right)}^{2}}$$

(21) $$L_{KSIso}^{S-}=\underset{Y}{\max}\sqrt{\sum_{(x_{i},x_{j})\in S^{-}}{\left(d_{G}\left(y_{i},y_{j}\right)-k\left(x_{i},x_{j}\right)\right)}^{2}}$$

To obtain the optimal solution for the above objective function, the problem is transformed to construct a more sensitive discriminative distance matrix. The specific steps of the KS-Isomap algorithm are as follows:

(1) Using the traditional Isomap algorithm, construct a simple undirected nearest neighbor graph, G. Then, calculate the geodesic distance, $d_{G}\left(x_{i},x_{j}\right)$, between data points $x_{i}$ and $x_{j}$ using the shortest path algorithm;
(2) Construct the discriminative distance matrix based on Equations (6) and (18):
  (22) $$\begin{array}{ll} D_{H} & =\left[d_{H}\left(x_{i},x_{j}\right)\right]\\ & =\left\{\begin{array}{cc} \sqrt{1-k_{Elinear}\left(x_{i},x_{j}\right)} & for\;L\left(x_{i}\right)=L\left(x_{j}\right)\\ \sqrt{k_{Elinear}\left(x_{i},x_{j}\right)}-\varphi & for\;L\left(x_{i}\right)\neq L\left(x_{j}\right) \end{array}\right. \end{array}$$
  And define
  $$D_{H}^{2}=\left\{\begin{array}{cc} \sqrt{1-k_{Elinear}^{2}\left(x_{i},x_{j}\right)} & for\;L\left(x_{i}\right)=L\left(x_{j}\right)\\ \sqrt{k_{Elinear}^{2}\left(x_{i},x_{j}\right)}-\varphi & for\;L\left(x_{i}\right)\neq L\left(x_{j}\right) \end{array}\right.$$
  where the parameter φ denotes the bias coefficient;
(3) Construct the following block matrix:
  (23) $$\left[\begin{array}{cc} 0 & 2D_{H}^{2}\\ -I & -4D_{H} \end{array}\right]$$
  And calculate its spectral radius, $\rho_{3}^{*}$;
(4) Construct the Mercer kernel matrix as the metric matrix for the third space based on the following equation:
  (24) $$K_{3}=K_{D}^{2}+2\rho_{3}^{m}K_{D}+\frac{1}{2}{\left(\rho_{3}^{m}\right)}^{2}H$$
  where the parameter $\rho_{3}^{m}\geq \rho_{3}^{*}$;
(5) Perform an eigenvalue decomposition and select the top $\lambda_{3}$ eigenvalues’ corresponding to the eigenvectors $\Gamma_{3}$ as the third piece of spatial feature information for $K_{3}$. Calculate the spectral radius $\rho_{3}$ of the first spatial metric matrix, $K_{3}$, to represent the first piece of spatial information.

### 3.3. AN-MFIDFS-Isomap Fault Diagnosis Arithmetric

**Figure 2:**
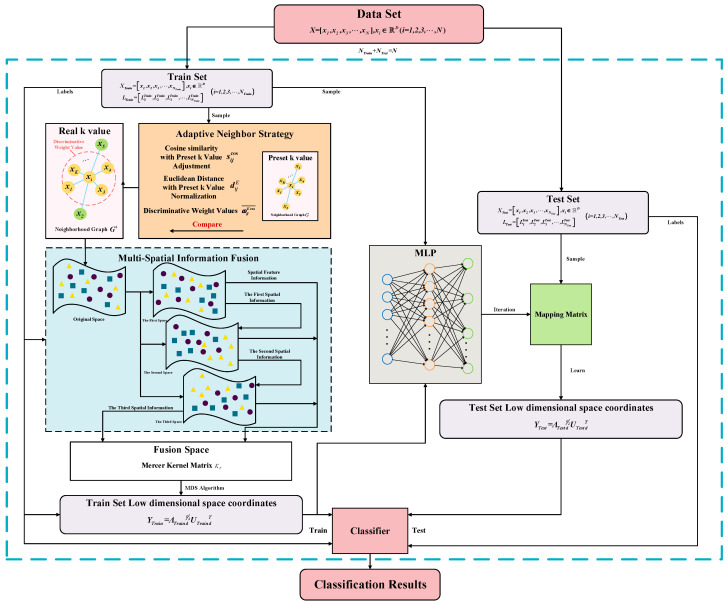
Flowchart of AN-MFIDFS-Isomap fault diagnosis arithmetic.

Currently, research on manifold learning algorithms is primarily confined to a high-dimensional Euclidean space or kernel Hilbert space, and the potential of leveraging feature information from multiple spaces corresponding to the same data has been overlooked. Therefore, this study, by utilizing the adaptive neighbor selection strategy proposed in Section 3.1 to obtain the metric matrix and employing the method proposed in Section 3.2 for feature space transformation and feature information extraction, presents a fault diagnosis algorithm called AN-MFIDFS-Isomap. The algorithm’s flowchart is illustrated in Figure 2, and the detailed steps of the algorithm are as follows:

(1) Divide the data into a training set, $X_{train}$, and a testing set, $X_{test}$, preprocess the data, and construct the corresponding label information for each set;
(2) For the training set, utilize the adaptive nearest neighbor strategy proposed in Section 3.1. Compute the geodesic distances under the rule of true nearest neighbors for the simple undirected neighbor graph, $G^{+}$, constructing the geodesic distance matrix, i.e., the metric matrix in the original space;
(3) Employ the first spatial transformation method proposed in Section 3.2.2 to obtain the first piece of spatial feature information, $\Gamma_{1}$, and the first piece of spatial information, $\rho_{1}$;
(4) Let the first piece of spatial information, $\rho_{1}$, be the linear weights, ϖ, from Equation (19), incorporating the first piece of spatial information into the second spatial transformation method to obtain the second spatial feature information, $\Gamma_{2}$, and the second spatial information, $\rho_{2}$;
(5) Let the second piece of spatial information, $\rho_{2}$, be the linear weights, ϖ of, the exponential linear kernel function from Equation (24), incorporating the first and second pieces of spatial feature information into the third spatial transformation method to obtain the third piece of spatial feature information, $\Gamma_{3}$. Linearly combine the feature information from the three spaces to obtain $\Gamma ={\left[\Gamma_{1},\Gamma_{2},\Gamma_{3}\right]}_{N*\left(\lambda_{1}+\lambda_{2}+\lambda_{3}\right)},\left(\sum \lambda_{i}=D,i=1,2,3\right)$ and define each element in the matrix $\Gamma^{2}$ as the square of the corresponding element in the matrix Γ.
(6) Construct the block matrix as follows:
  (25) $$\left[\begin{array}{cc} 0 & 2\Gamma^{2}\\ -I & -4\Gamma \end{array}\right]$$
  And calculate its spectral radius, $\rho^{*}$. Then, construct the Mercer kernel matrix according to the following equation, which serves as the metric matrix for the fused space:
  (26) $$K_{F}=\Gamma^{2}+2\rho^{m}\Gamma +\frac{1}{2}{\left(\rho^{m}\right)}^{2}H$$
  where the parameter $\rho^{m}\geq \rho^{*}$;
(7) Apply classical MDS to compute the low-dimensional embedded manifold coordinates $Y_{train}$ for the training set using the metric matrix $K_{F}$;
(8) Train a classifier using the low-dimensional embedded coordinates, Y, and their corresponding label information. Simultaneously, use a Multi-Layer Perceptron (MLP) to iteratively obtain the mapping matrix from the original training set, X, to the low-dimensional embedded manifold coordinates, Y;
(9) Multiply the test set $X_{test}$ by the mapping matrix obtained through the MLP iterations to obtain the low-dimensional embedded manifold coordinates for the test set, denoted as $Y_{test}$;
(10) Perform fault diagnosis on the low-dimensional embedded manifold coordinates for the test set $Y_{test}$ using the trained classifier.

The three indicators in Fisher’s discriminant criterion, the inter-class scatter, $S_{\mathrm{inter}}$, the intra-class scatter, $S_{\mathrm{intra}}$, and Fisher’s metric, *F*, help calculate the distances and separation level between data points in the low-dimensional space, describing the clustering effectiveness of the dimensionality reduction algorithm. The definitions of these three indicators are as follows:

(27) $$S_{\mathrm{inter}}=\sum_{i=1}^{L}\left(\bar{x_{i}}-\bar{x}\right){\left(\bar{x_{i}}-\bar{x}\right)}^{T}$$

(28) $$S_{\mathrm{intra}}=\sum_{i=1}^{L}\sum_{j=1}^{N_{i}}\left(x_{i}^{j}-\bar{x_{i}}\right){\left(x_{i}^{j}-\bar{x_{i}}\right)}^{T}$$

(29) $$F=\frac{w^{T}S_{\mathrm{inter}}w}{w^{T}S_{i\mathrm{ntra}}w}$$

where w denotes all the one-column vectors, $\bar{x_{i}}$ represents the mean vector for the *i*-th class, $\bar{x}$ represents the overall mean vector for all the sample points, $x_{i}^{j}$ represents the *j*-th sample point in the *i*-th class, and L represents the number of classes. The confusion matrix records the complete results of the fault diagnosis, where the rows represent predicted labels and the columns represent true labels.

For the classification algorithm performance, we record the diagnostic process in detail using a confusion matrix and evaluate it using evaluation indexes such as accuracy, precision, recall, and the *F*1 score with the following expressions:

(30) $$Accuracy=\frac{TP+TN}{TP+FP+TN+FN}$$

(31) $$Precision=\frac{TP}{TP+FP}$$

(32) $$Recall=\frac{TP}{TP+FN}$$

(33) $$F1\_score=2\times \frac{Precision\times Recall}{Precision+Recall}$$

where *TP* denotes a true positive and *TN* denotes a true negative. *FP* denotes the false positive, and *FN* denotes a false negative.

## 4. Experiment and Analysis

In this section, the effectiveness of the proposed algorithm is evaluated using two cases in the context of rolling bearings, which are essential components of rotating machinery. The diagnostic results are compared with eight other commonly used manifold learning algorithms. The experiments were conducted using Python 3 and PyCharm 2021 software, PyCharm Community Edition 2021.3.2, running on hardware equipped with an 11-th Gen Intel(R) Core(TM) i7-11850H CPU @ 2.50GHz.

### 4.1. Case 1: CWRU

#### 4.1.1. Data Description

**Figure 3:**
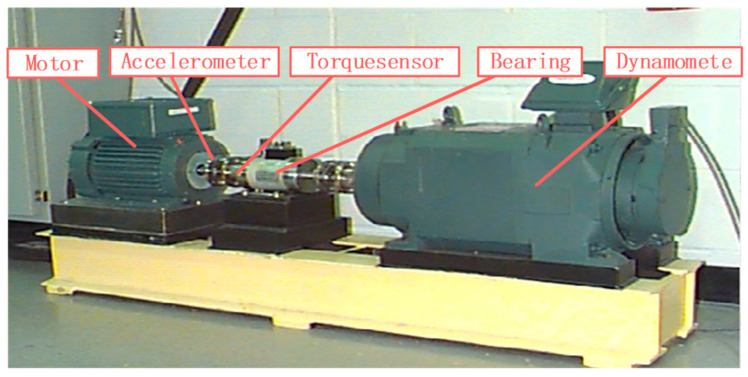
The bearing test rig of CWRU.

The vibration data used in this case study are sourced from the Case Western Reserve University (CWRU) Bearing Data Center, as depicted in Figure 3. The experimental setup comprises components such as a motor, accelerometer, torque sensor, bearing, and dynamometer. The data collection involved a deep groove ball bearing SFK 6205, with bearing health states including Normal (NO), Rolling Element Fault (RF), Inner Race Fault (IF), and Outer Race Fault (OF), totaling four distinct modes [35].

**Figure 4:**
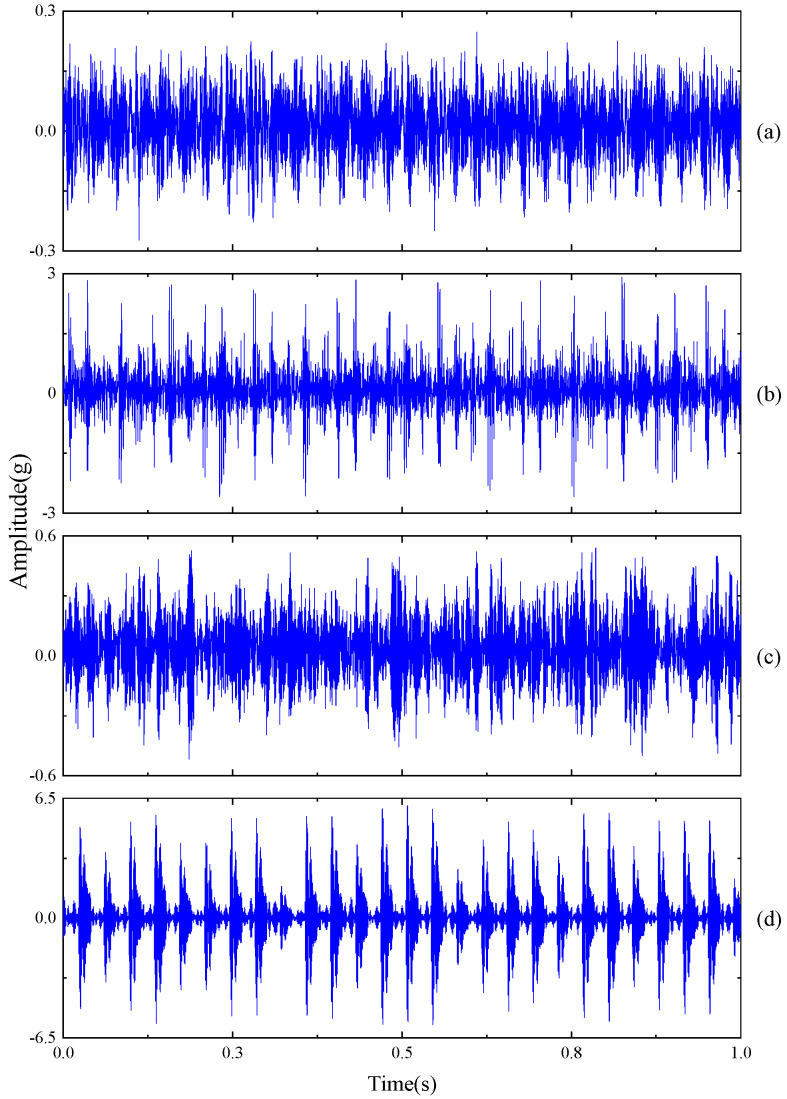
Vibration signals of four work conditions for Case 1: (**a**) NO; (**b**) IF; (**c**) RF; (**d**) OF.

The data in this case study were collected using an accelerometer, with the measurement point located at the motor drive end. The sampling frequency was set at 12 kHz. Detailed information regarding the rolling bearings is presented in Table 1. Additionally, the original vibration signals are depicted in Figure 4.

The experimental data were resampled to select 160 samples for each operational state of the bearing as the training set. The sampling window had a length of 1024, and the sliding window size was set at 512. For each sample, 17 time-domain features and 12 frequency-domain features were extracted. The same methodology was applied to select 40 samples for each operational state of the bearing as for the testing set.

#### 4.1.2. Model Parameters Setting and Implementation

To comprehensively evaluate the proposed method, it is compared with eight other algorithms: PCA, MDS, Isomap, LE, LLE, HLLE, LTSA, and T-SNE. Among them, PCA and MDS are two commonly used linear manifold learning algorithms that have shown good performance in fault diagnosis applications. Isomap is a classic algorithm for preserving the global embedding in manifold learning, and LLE is a classic algorithm for preserving the local embedding. LE, HLLE, and LTSA have been improved by researchers based on LLE and have shown good performance in dimensionality reduction.

The detail parameter descriptions of all the methods are listed in Table 2.

To ensure the completeness of the experiments, we used six different classifiers: a logistic regression, a decision tree, a random forest, a plain Bayes, k-nearest neighbors (KNNs), and a support vector machine (SVM). These classifiers were trained using the dimensionality-reduced data obtained from various methods. Subsequently, the trained classifiers were tested using the test set.

For each method, we applied the dimensionality reduction technique and then fed the reduced data into each of the six classifiers mentioned above. The classifiers were trained using the training data, and their performance was evaluated using the test data.

#### 4.1.3. Diagnosis Results and Discussion

We conducted two comprehensive experiments to compare our proposed method with others.

In the first experiment, we applied all the experimental methods to perform a dimensionality reduction on the data and conducted a quantitative analysis of clustering capability using Fisher’s discriminant criterion. In the second experiment, we separately trained different classifiers using the reduced data obtained from each method and conducted a quantitative analysis of the model’s diagnostic accuracy.

**Figure 5:**
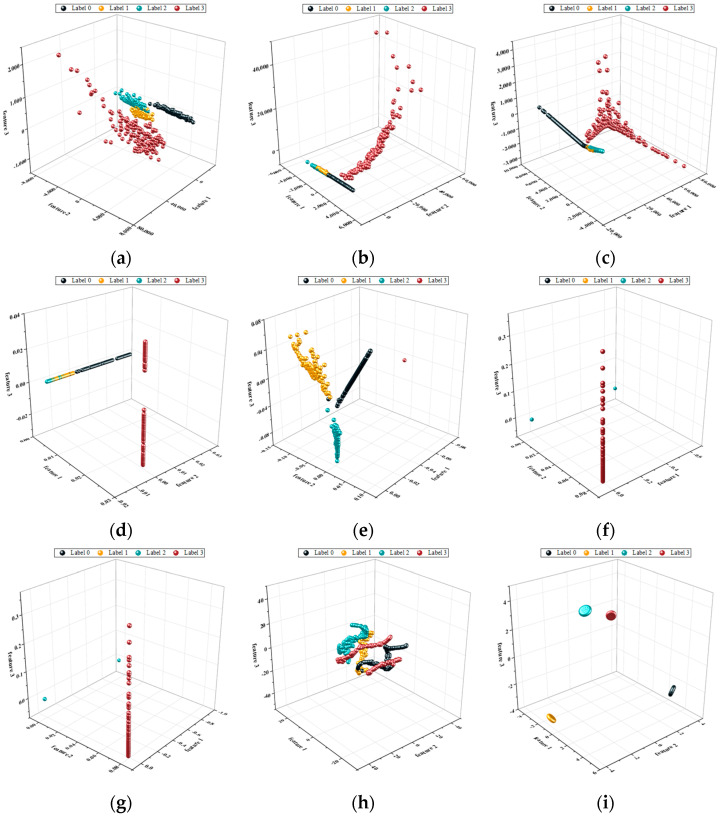
Scatter plots of low-dimensional features in 3D feature space for Case 1: (**a**) PCA; (**b**) MDS; (**c**) Isomap; (**d**) LE; (**e**) LLE; (**f**) HLLE; (**g**) LTSA; (**h**) T-SNE; (**i**) proposed method.

In the first experiment, to intuitively demonstrate the superiority of our method in terms of its clustering capability, we applied our proposed method and eight other methods to perform a dimensionality reduction on the vibration data from different states of the bearing. In Figure 5, we present 3D scatter plots of the abstract feature space of the bearing data after the dimensionality reduction.

From Figure 5a–h, it can be observed that after the dimensionality reduction using the traditional manifold learning algorithms, there are varying degrees of overlap between samples of different categories in the 3D feature space. Conversely, after the dimensionality reduction, using our proposed method, the data in the 3D feature space exhibit a better separation. This demonstrates the strong clustering capability achieved through the fusion of multi-feature information.

To provide a more precise description of our method’s clustering capability, we calculated Fisher’s discriminant criterion for the dimensionality-reduced data from all the methods. Table 3 presents detailed indicators of the Fisher criterion for all the methods. From Table 3, it can be observed that our method has the largest between-class scatter and the smallest within-class scatter. Although our method does not have the maximum class separability (Fisher’s criterion), it does have the largest Fisher measure (*F*), indicating that using our method for dimensionality reduction makes it easier to distinguish between data from different categories. This once again demonstrates the strong clustering capability of our method.

**Figure 6:**
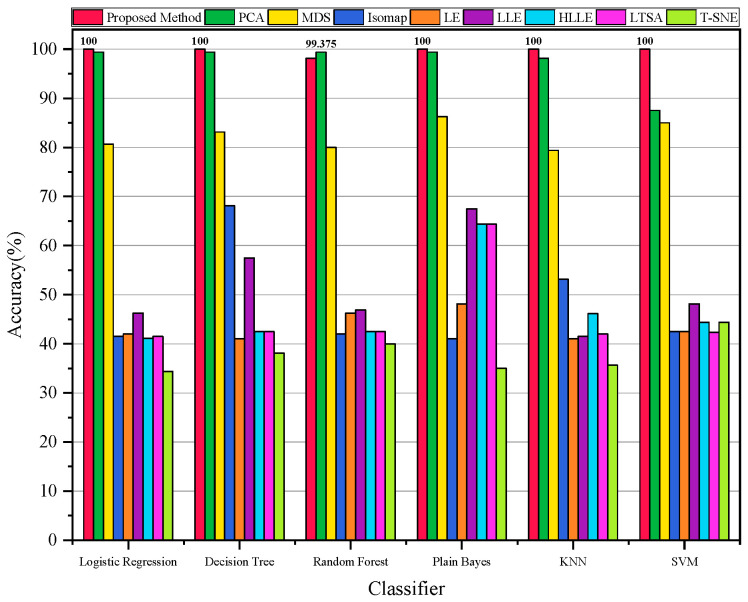
Accuracy of all methods with different classifiers for Case 1.

In the second experiment, to demonstrate the better diagnostic accuracy of our algorithm, we applied our proposed method and eight other methods to diagnose the vibration data from different states of the bearing. Figure 6 illustrates the diagnostic accuracy of all the methods when using different classifiers. It can be observed that our proposed method exhibits high accuracy across various classifiers, reaching a maximum classification accuracy of 100%.

**Figure 7:**
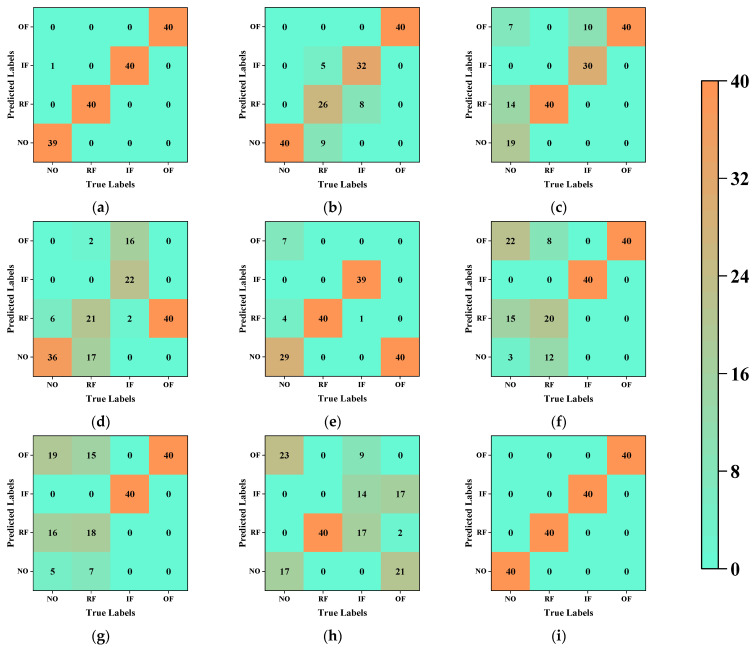
Confusion matrices of all method for Case 1: (**a**) PCA–Random Forest; (**b**) MDS–Decision Tree; (**c**) Isomap–Plain Bayes; (**d**) LE–Plain Bayes; (**e**) LLE–Plain Bayes; (**f**) HLLE–Plain Bayes; (**g**) LTSA–Plain Bayes; (**h**) T-SNE–SVM; (**i**) proposed method–SVM.

We used a confusion matrix to document in detail the diagnostic process with the highest accuracy for the different fault diagnostic methods. Figure 7 presents the detailed contents of the confusion matrices. Evidently, most misclassified samples have overlapping fault characteristics, thus reducing the distinctiveness between the different categories of samples. Table 4 records the evaluation indexes at the best accuracy of each type of fault diagnosis method. It can be seen that from the data in Case 1 that our proposed method achieves good performance on all evaluation indexes.

### 4.2. Case 2: Laboratory-Built Bearing Experimental Rig

#### 4.2.1. Data Description

**Figure 8:**
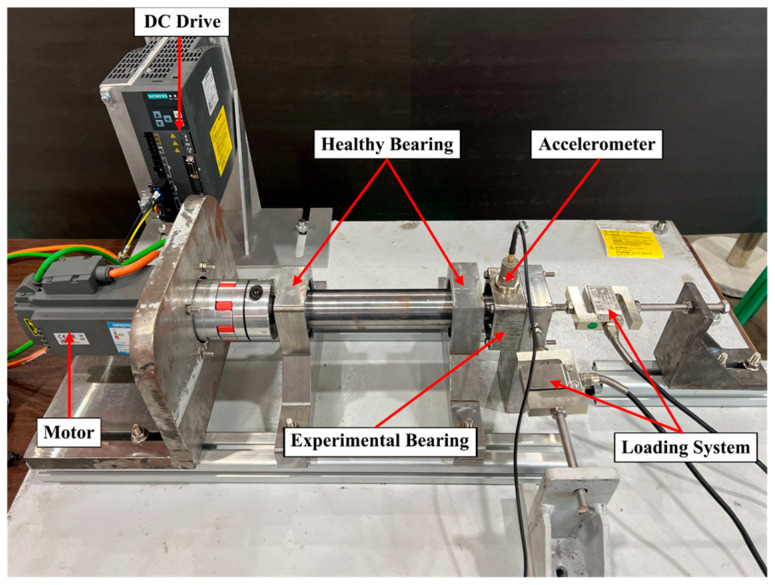
The laboratory-built bearing experimental rig.

**Figure 9:**
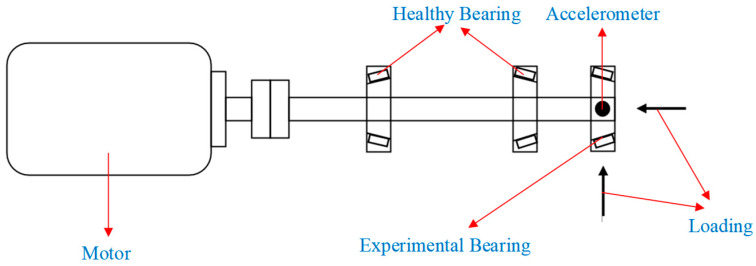
The working schematic of laboratory-built bearing experimental rig.

**Figure 10:**
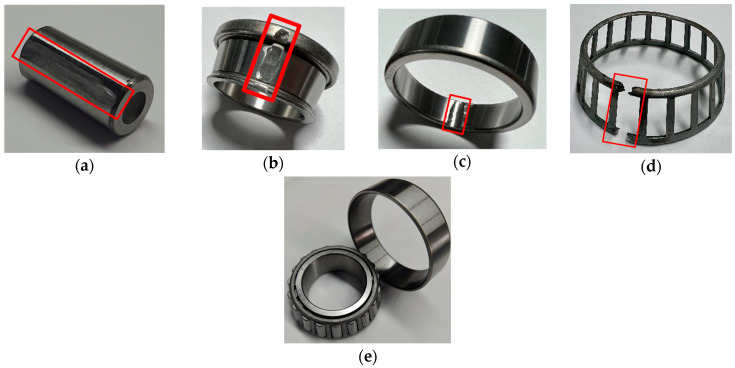
Bearing health status: (**a**) Rolling Element Fault; (**b**) Inner Race Fault; (**c**) Outer Race Fault; (**d**) Cage Fault; (**e**) Healthy Bearing. The red box shows the exact shape of the bearing fault in detail.

The bearing vibration dataset used in this case study was obtained from a laboratory-built experimental rig. The laboratory-built bearing experimental rig and its theory graph are illustrated in Figure 8 and Figure 9, respectively. The setup comprises components such as a motor, DC driver, healthy bearing, experimental bearing, accelerometer, and loading system. The data collection was performed on a tapered roller bearing, model 33007. The bearing’s health conditions encompass five modes, Normal (NO), Rolling Element Fault (RF), Inner Race Fault (IF), Outer Race Fault (OF), and Cage Fault (CF), as depicted in Figure 10.

**Figure 11:**
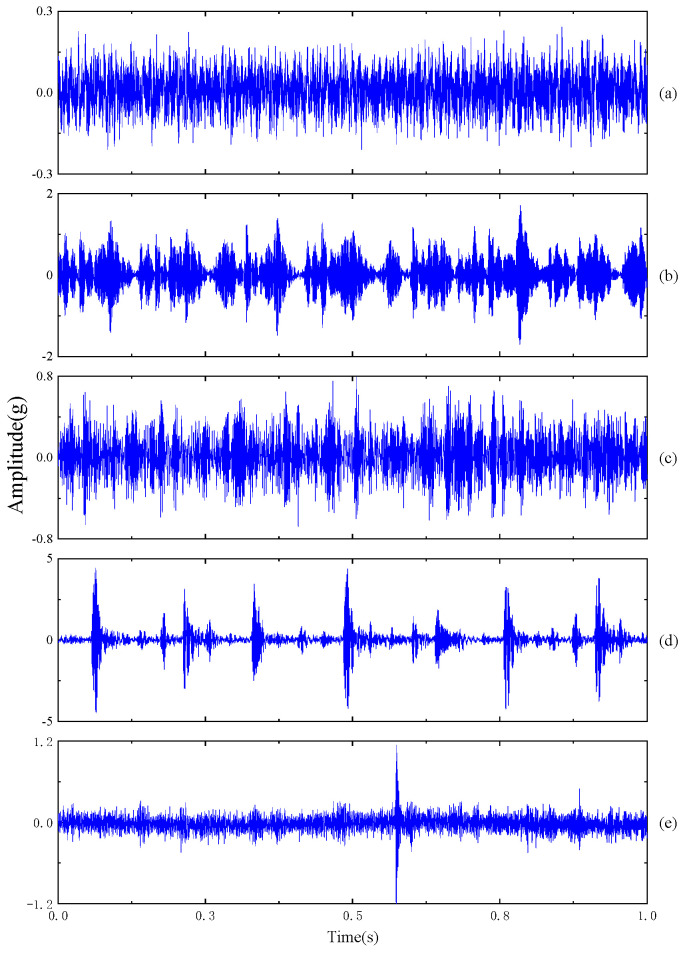
Vibration signals of five work conditions for Case 2: (**a**) NO; (**b**) IF; (**c**) RF; (**d**) OF; (**e**) CF.

The data used in this case were collected with an accelerometer, located at the testing bearing position, with a sampling frequency of 12 kHz. The motor speed was 600 rpm, and the axial and radial loads were 1 kN. This case focuses on diagnosing the vibration data of rolling bearings under bi-directional loads. Detailed information about the rolling bearing is provided in Table 5, and the original vibration signals are depicted in Figure 11. The experimental data were preprocessed using the same methods as in Case 1.

#### 4.2.2. Model Parameters Setting and Implementation

To comprehensively evaluate the performance of the proposed method on the laboratory-built experimental rig, we continue to utilize a grid search to select the optimal hyperparameters for each algorithm. The detailed parameter descriptions for all the methods are provided in Table 6. This is to substantiate what was mentioned in Section 3.2.3.

#### 4.2.3. Diagnosis Results and Discussion

We conduct two experiments, similar to Case 1, to comprehensively compare the performance of our proposed method and other methods on the laboratory-built experimental rig.

In the first experiment, we apply all experimental methods to perform a dimensionality reduction on the data and quantitatively analyze the clustering ability using Fisher’s discriminant criterion. In the second experiment, we train different classifiers separately using the dimensionally reduced data obtained by each method and analyze the model’s diagnostic accuracy. Additionally, in the third experiment, we use a Gaussian kernel function, a linear kernel function, and the proposed exponential linear kernel function to construct the second spatial matrix for the dimensionality reduction. We then quantify the model’s diagnostic accuracy using the best-performing classifier.

**Figure 12:**
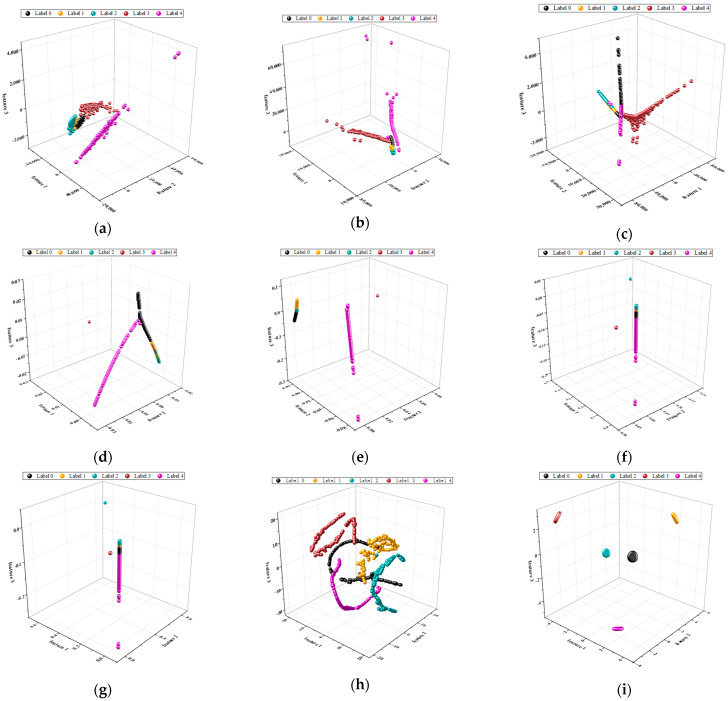
Scatter plots of low-dimensional features in 3D feature space for Case 2: (**a**) PCA; (**b**) MDS; (**c**) Isomap; (**d**) LE; (**e**) LLE; (**f**) HLLE; (**g**) LTSA; (**h**) T-SNE; (**i**) proposed method.

In the first experiment, Figure 12 visually demonstrates the superiority of our method in terms of its clustering ability. As observed in Figure 12a–h, the traditional manifold learning algorithms still exhibit varying degrees of overlap in the case of Case 2. On the other hand, our proposed method continues to perform well, showcasing its robust clustering ability when applied to different datasets.

To provide a more precise description of our method’s clustering ability, we calculate Fisher’s discriminant criterion for the dimensionally reduced data obtained by all the methods. Table 6 presents the detailed metrics based on the Fisher discriminant criterion for each method. A comparison between Table 3 and Table 7 reveals an overall improvement in the *F* values for all the methods. Our proposed method maintains the highest *F* value, indicating excellent discriminability between different categories of data in Case 2 after using our method for dimensionality reduction. This once again strongly validates the robust clustering ability of our proposed method.

**Figure 13:**
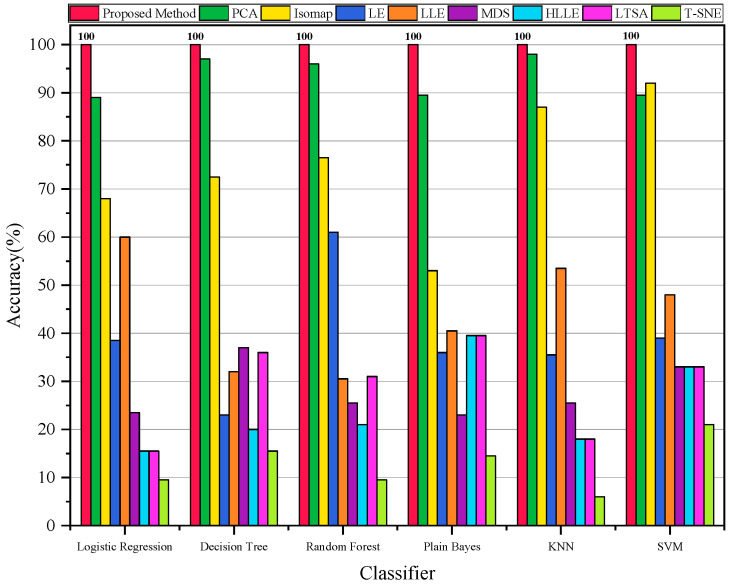
Accuracy of all methods with different classifiers for Case 2.

In the second experiment, to validate the superior performance of our algorithm in terms of its diagnostic accuracy, we applied our proposed method as well as eight other methods to diagnose faults in the Case 2 bearing vibration data. Figure 13 illustrates the diagnostic accuracy of all the methods when using different classifiers. It is evident that our proposed method exhibits higher stability in terms of diagnostic accuracy across various classifiers, with the highest accuracy reaching 100%.

**Figure 14:**
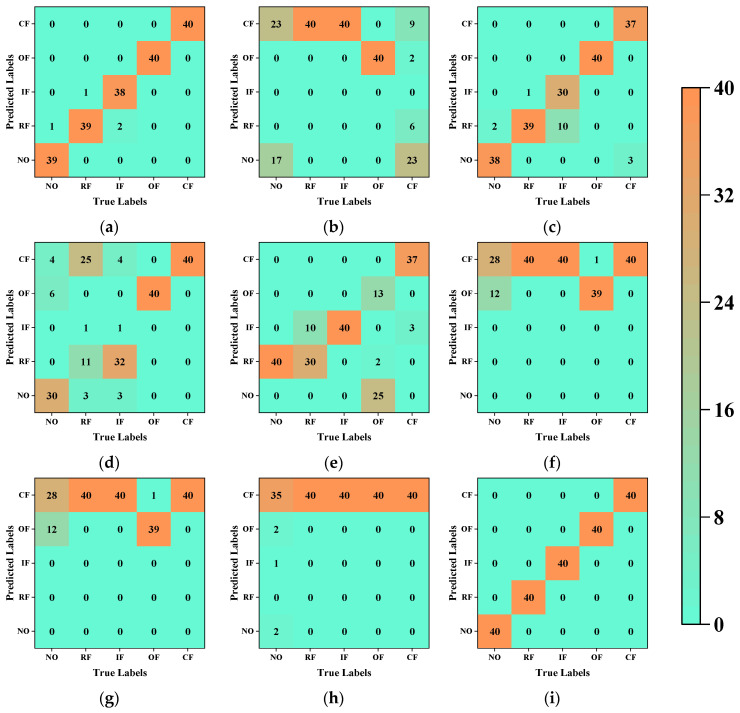
Confusion matrices of all method for Case 2: (**a**) PCA–Decision Tree; (**b**) MDS–Decision Tree; (**c**) Isomap–SVM; (**d**) LE–Random Forest; (**e**) LLE–Logistic Regression; (**f**) HLLE–Plain Bayes; (**g**) LTSA–Plain Bayes; (**h**) T-SNE–SVM; (**i**) proposed method–SVM.

For the fault diagnosis, we utilized the classifier that demonstrated the best accuracy for each method and recorded the diagnostic process using confusion matrices. Figure 14 provides detailed insights into the confusion matrices. Table 8 records the evaluation indexes at the best accuracy of each type of fault diagnosis method. It can be seen that for the data in Case 2, our proposed method achieves better performance on all the evaluation indexes than any other method.

**Figure 15:**
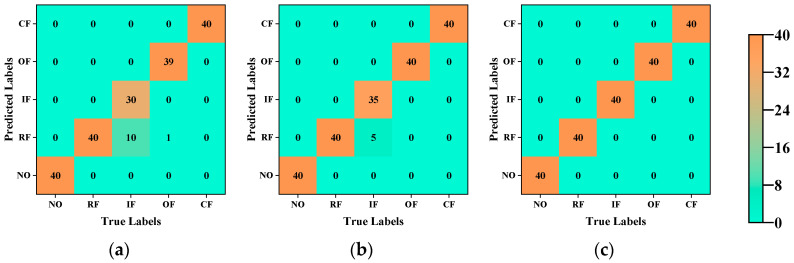
Confusion matrix of different kernel functions for Case 2: (**a**) linear kernel function; (**b**) gaussian kernel function; (**c**) exponential linear kernel function.

In the third experiment, to showcase the superiority of our proposed kernel function over the traditional ones, we employed a linear kernel, a Gaussian kernel, and our proposed exponential linear kernel to extract features in the second space. Figure 15 illustrates confusion matrices detailing fault diagnosis using different kernel functions for feature extraction in the second space. It is evident that our proposed exponential linear kernel can extract more effective feature information, resulting in higher diagnostic accuracy.

## 5. Conclusions

Addressing the issue of dimensionality is a challenge in the current field of intelligent fault diagnosis of rolling bearings, so this paper proposes a supervised manifold learning method that integrates multiple pieces of feature information for diagnosing rolling bearing faults. Firstly, an adaptive nearest neighbor strategy is employed to reconstruct the manifold neighbor graph. Subsequently, multiple spatial transformation techniques are introduced to acquire feature information in different spaces. Notably, an innovative exponential linear kernel function and the KS-Isomap algorithm are presented to enrich the feature space with novel information. The multi-space feature information is then fused with discriminative information derived from the data labels, leading to the development of a supervised manifold learning method for feature extraction. Finally, this method is employed in collaboration with classifiers to conduct fault diagnosis on rolling bearings. The experimental validation using the CWRU open dataset and our laboratory-built experimental data demonstrates that the proposed AN-MSDIS-Isomap algorithm outperforms traditional manifold learning methods in clustering, dimension reduction, and fault diagnosis. It exhibits consistently good classification accuracy across various classifiers, with the highest classification accuracy reaching 100%.

The proposed method addresses the challenge of dimensionality and effectively extracts significant features representing the data. When combined with a classifier, it performs well in fault diagnosis tasks. Future research will focus on fault diagnosis tasks for bearing vibration signals in strong noise environments and the optimization of fault diagnosis performance between algorithms and different classifiers. Meanwhile, aiming to solve the problem of the high computational complexity of machine learning algorithms, research on methods to reduce computational costs and improve computational efficiency should be conducted.

## 6. Patents

There is a patent named fault diagnosis methods for mechanical equipment (patent number: ZL 2023 1 0839479.7) resulting from the work reported in this manuscript.

## Figures and Tables

**Table 1:** Different work conditions of drive end bearings details.

Patterns	Labels
NO	0
IF	1
RF	2
OF	3

**Table 2:** Parameter settings of all methods in Case 1.

Methods	Parameters Settings
Proposed method	Intrinsic dimension d = 4, the number of nearest neighbors k = 10, the offset coefficient b = 0.5, the bias coefficient φ = 0.5. The structure of MPL is 29-128-256-128-4 for the training data. The learning rate = 0.001; the iteration number of pre-training is 1000. The optimizer is Adam.
PCA	Intrinsic dimension d = 4.
MDS	Intrinsic dimension d = 4.
Isomap	Intrinsic dimension d = 5; the number of nearest neighbor k = 5.
LE	Intrinsic dimension d = 4; the number of nearest neighbor k = 5.
LLE	Intrinsic dimension d = 2; the number of nearest neighbor k = 30.
HLLE	Intrinsic dimension d = 3; the number of nearest neighbor k = 25.
LTSA	Intrinsic dimension d = 9; the number of nearest neighbor k = 30.
T-SNE	Intrinsic dimension d = 2; perplexity *p* = 30.

**Table 3:** The detailed Fisher statistical quantity of all methods for Case 1.

Methods	$S_{\mathrm{inter}}$	$S_{\mathrm{intra}}$	*F*
Proposed method	2.5009 × 10^4^	3.6600 × 10	6.8331 × 10^2^
PCA	6.3453 × 10^10^	3.0215 × 10^10^	2.1000
MDS	6.3283 × 10^10^	3.0406 × 10^10^	2.0812
Isomap	8.9909 × 10^10^	3.2154 × 10^10^	2.7962
LE	1.6468 × 10^−1^	1.12566 × 10^−1^	1.3106
LLE	2.5500	2.0001 × 10^−1^	1.2749 × 10
HLLE	8.9230 × 10^−1^	1.4363	6.2125 × 10^−1^
LTSA	7.86899 × 10^−1^	1.8633	4.2231 × 10^−1^
T-SNE	7.2028 × 10^4^	1.3775 × 10^5^	5.2287 × 10^−1^

**Table 4:** The detailed evaluation indexes of all methods with the best accuracy for Case 1.

Methods	Accuracy	Precision	Recall	F1_Score
Proposed method	100%	100%	100%	1
PCA	99.375%	97.5%	100%	0.9873
MDS	86.25%	96.5035%	89.0323%	0.9261
Isomap	68.125%	83.8462%	78.4173%	0.8104
LE	49.375%	76.6990%	57.2464%	0.6556
LLE	67.5%	90.7563%	72.4832%	0.8060
HLLE	64.375%	69.5946%	89.5652%	0.7833
LTSA	64.375%	67.3203%	93.6364%	0.7833
T-SNE	44.375%	68.9320%	56.3492%	0.6201

**Table 5:** Different work conditions of laboratory-built bearing experimental rig details.

Patterns	Fault Diameter (mm)	Labels
NO	0	0
IF	13.8 × 4.8	1
RF	13.5 × 3.2	2
OF	16.6 × 4.7	3
CF	16.7 × 4.3	4

**Table 6:** Parameter settings of all methods in Case 2.

Methods	Parameters Settings
Proposed method	Intrinsic dimension d = 3, the number of nearest neighbor k = 10, the offset coefficient b = 0.5, the bias coefficient φ = 0.5. The structure of MPL is 29-128-256-128-5 for the training data. The learning rate = 0.001; the iteration number of pre-training is 1000. The optimizer is Adam.
PCA	Intrinsic dimension d = 4.
MDS	Intrinsic dimension d = 4.
Isomap	Intrinsic dimension d = 4; the number of nearest neighbor k = 15.
LE	Intrinsic dimension d = 3; the number of nearest neighbor k = 25.
LLE	Intrinsic dimension d = 4; the number of nearest neighbor k = 15.
HLLE	Intrinsic dimension d = 3; the number of nearest neighbor k = 25.
LTSA	Intrinsic dimension d = 7; the number of nearest neighbor k = 25.
T-SNE	Intrinsic dimension d = 4; perplexity *p* = 15.

**Table 7:** The detail Fisher statistical quantity of all methods for Case 2.

Methods	$S_{\mathrm{inter}}$	$S_{\mathrm{intra}}$	*F*
Proposed method	4.2742 × 10^4^	3.8155 × 10	1.1202 × 10^3^
PCA	1.2840 × 10^11^	5.7755 × 10^10^	2.2232
MDS	1.2842 × 10^11^	5.7817 × 10^10^	2.2211
Isomap	2.0071 × 10^11^	6.5189 × 10^10^	3.0789
LE	2.7861 × 10^−1^	4.0982 × 10^−2^	6.7985
LLE	1.9852	8.1480 × 10^−1^	2.4364 × 10
HLLE	1.5833	5.0543 × 10^−1^	3.1325
LTSA	1.1178	1.3131	8.5121 × 10^−1^
T-SNE	1.2986 × 10^5^	1.0083 × 10^5^	1.2879

**Table 8:** The detailed evaluation indexes of all methods with the best accuracy for Case 2.

Methods	Accuracy	Precision	Recall	F1_Score
Proposed method	100%	100%	100%	1
PCA	98%	98.9899%	98.9899%	0.9899
Isomap	92%	98.3957%	93.4010%	0.9583
LE	61%	75.3086%	76.25%	0.7578
LLE	60%	70.5882%	80%	0.75
MDS	33%	39.0533%	69.4737%	0.5
HLLE	39.5%	39.5%	100%	0.5663
LTSA	39.5%	39.5%	100%	0.5663
T-SNE	21%	21%	100%	0.3471

## Data Availability

The data presented in this study are available on request from the corresponding author. The data are not publicly available due to the grant program has not yet been completed.

## Appendix A

### Proof of Semi-Positive Definite Property to Exponential Linear Kernel Function Matrix

According to the definition of the exponential linear kernel function in Section 3.2.3, it is evident that it incorporates distance information, rendering the exponential linear kernel function matrix a symmetric matrix. Subsequently, we only need to demonstrate its positive semi-definite property to establish the exponential linear kernel function as a valid kernel function. The expression for the exponential linear kernel function matrix is as follows:

(A1) $$K_{Elinear}=\left[k_{Elinear}\left(x_{i},x_{j}\right)\right]=\left[e^{-\left(\varpi d_{E}\left(x_{i},x_{j}\right)+b\right)}\right]$$

The proof of the positive semi-definite property is presented as follows:

(A2) $$e^{-\left(\varpi d_{E}\left(x_{i},x_{j}\right)+b\right)}$$

(A3) $$=e^{-\left(\varpi {\left\|x_{i}-x_{j}\right\|}^{2}+b\right)}$$

(A4) $$=e^{-b}\cdot e^{-\varpi {\left\|x_{i}-x_{j}\right\|}^{2}}$$

(A5) $$=e^{-b}\cdot e^{-\varpi \sum_{k=1}^{D}{\left(x_{i}^{\left(k\right)}\right)}^{2}}\cdot e^{-\varpi \sum_{k=1}^{D}{\left(x_{j}^{\left(k\right)}\right)}^{2}}\cdot \prod_{k=1}^{D}e^{2\varpi x_{i}^{\left(k\right)}x_{j}^{\left(k\right)}}$$

By performing a Taylor expansion on the fourth term of Equation (A4), we obtain:

(A6) $$=e^{-b}\cdot e^{-\varpi \sum_{k=1}^{D}{\left(x_{i}^{\left(k\right)}\right)}^{2}}\cdot e^{-\varpi \sum_{k=1}^{D}{\left(x_{j}^{\left(k\right)}\right)}^{2}}\cdot \prod_{k=1}^{D}\sum_{m=0}^{\infty }\left[\frac{{\left(2\varpi \right)}^{m}}{m!}{\left(x_{i}^{\left(k\right)}\right)}^{m}{\left(x_{j}^{\left(k\right)}\right)}^{m}\right]$$

(A7) $$=\sum_{m=0}^{\infty }e^{-\varpi \sum_{k=1}^{D}{\left(x_{i}^{\left(k\right)}\right)}^{2}}\cdot e^{-b/2}\cdot \prod_{k=1}^{D}\left[\sqrt{\frac{{\left(2\varpi \right)}^{m}}{m!}}{\left(x_{i}^{\left(k\right)}\right)}^{m}\right]\cdot \sum_{m=0}^{\infty }e^{-\varpi \sum_{k=1}^{D}{\left(x_{j}^{\left(k\right)}\right)}^{2}}\cdot e^{-b/2}\cdot \prod_{k=1}^{D}\left[\sqrt{\frac{{\left(2\varpi \right)}^{m}}{m!}}{\left(x_{j}^{\left(k\right)}\right)}^{m}\right]$$

(A8) $$={\left\|\sum_{i=1}^{N}\Phi \left(x_{i}\right)\right\|}^{2}\geq 0$$

$\Phi \left(x_{i}\right)=\sum_{m=0}^{\infty }e^{-\varpi \sum_{k=1}^{D}{\left(x_{i}^{\left(k\right)}\right)}^{2}}\cdot e^{-b/2}\cdot \prod_{k=1}^{D}\left[\sqrt{\frac{{\left(2\varpi \right)}^{m}}{m!}}{\left(x_{i}^{\left(k\right)}\right)}^{m}\right]$.

Therefore, it is evident that the exponential linear kernel function satisfies the necessary and sufficient conditions for being a kernel function.
